# Toward transmasculine-inclusive addiction psychiatry in India: a call for research

**DOI:** 10.3389/fpsyt.2025.1730507

**Published:** 2026-01-09

**Authors:** Anithamol Babu, Kalpana Sarathy, Aanandh C. Rajappan, Akhil P. Joseph

**Affiliations:** 1 Marian College Kuttikkanam Autonomous, Kuttikkanam, India; 2 Tata Institute of Social Sciences Guwahati, Jalukbari, Assam, India; 3 Amigos Trans Collective, Ernakulam, Kerala, India

**Keywords:** addiction psychiatry, community-based research, gender-affirming care, health policy, India, minority stress, substance use disorders, transmasculine health

Despite progressive policy frameworks such as the Transgender Persons (Protection of Rights) Act (2019) and the National Mental Health Programme, addiction psychiatry in India continues to neglect transmasculine individuals. Although “transgender inclusion” has become a normative policy ideal, the specificity of trans men’s experiences is rarely recognised in either clinical practice or academic research (1). This erasure is structural rather than accidental, with profound consequences that perpetuate cycles of distress and foreclose equitable access to care. The problem begins with data itself. National surveys led by the National AIDS Control Organisation (NACO) and the Ministry of Health and Family Welfare continue to collapse all transgender experiences into a single category, obscuring the distinct vulnerabilities of transmasculine populations (2). The lack of disaggregated data masks substance use patterns, comorbid health outcomes, and psychiatric risks specific to trans men. Empirical evidence from India indicates that among transgender individuals, alcohol and tobacco use disorders are the most prevalent forms of addiction, with illicit drug use and polysubstance use also documented, and community-based studies report wide variation with alcohol abuse ranging from 23% to 62.5% and dependence reaching approximately 31.2% (3). International evidence similarly shows elevated substance use among transgender and gender-diverse populations, with meta-analytic data indicating substantially higher odds of use globally, including past-month illicit drug use among transgender young adults at roughly 24.7% compared to 5.1% among cisgender peers (4, 5). By treating “transgender” as a monolith, health systems not only fail to capture epidemiological realities but also reinforce diagnostic opacity that prevents the development of tailored interventions. In reality, “transgender” refers to a heterogeneous set of identities—including trans women, trans men, nonbinary people, hijras, and other gender-diverse groups—yet most Indian research focuses almost exclusively on trans women and hijra communities, leaving trans men, nonbinary persons, and other subgroups largely absent from empirical literature. Consequently, existing prevalence estimates cannot be assumed to represent all transgender populations, as epidemiological patterns remain profoundly limited without subgroup-specific data. This is an Indian anomaly, one that contrasts with global frameworks such as the UNDP LGBTI Inclusion Index (6), which stresses disaggregation as the foundation of inclusive health governance. Without such specificity, addiction psychiatry cannot meaningfully identify, let alone address, the health needs of transmasculine people.

The current evidence base highlights stark research gaps. India-specific data on transmasculine mental health and substance use are almost non-existent. Most available studies focus on transfeminine populations or transgender groups, masking trans men’s experiences. Studies of trans women in India show high rates of mental health challenges and some high-risk alcohol use, particularly among sex workers (7–9). International evidence is more illuminating: transmasculine adults in the United States report extremely high rates of adverse childhood experiences (ACEs), with over 90% reporting at least one, strongly linked to depression, PTSD, suicidality, intimate partner violence, obesity, and substance use (10). Among youth, transmasculine individuals face disproportionately high risks, with studies showing 62% reporting depression and significantly higher self-harm and suicidal ideation compared to cisgender peers (11). These findings underscore the urgency of generating India-specific evidence to inform inclusive addiction psychiatry. Importantly, India has seen some promising but limited initiatives in transgender health, such as NACO’s inclusion of transgender populations in HIV programs and a few NGO-led harm reduction projects. Yet these rarely distinguish transmasculine needs, pointing to the gap between generalized inclusion efforts and identity-specific care.

Clinical practices compound invisibility in significant ways. India’s addiction treatment remains anchored in abstinence-based models, detoxification protocols, and brief counselling approaches, frameworks designed primarily for cisgender men (12). At the level of therapeutic modalities, mainstream services in India encompass a wide spectrum of interventions (13–15). Medical detoxification constitutes the initial phase for individuals with physiological dependence—especially on alcohol, opioids, or sedative-hypnotics—through medically supervised withdrawal management and the use of pharmacotherapies such as benzodiazepines for alcohol withdrawal, opioid agonist or antagonist therapies, and relapse-prevention agents including disulfiram, naltrexone, and acamprosate. This phase stabilises neurochemical states and creates a foundation for psychosocial work. Following detoxification, psychosocial therapies—including individual and group counselling, psychoeducation, cognitive-behavioural therapy, motivational interviewing, relapse-prevention planning, and stress-management approaches—address psychological, relational, and behavioural drivers of substance use.

In moderate to severe cases, structured residential rehabilitation provides a controlled therapeutic milieu with integrated medical, psychological, and social interventions, daily routines, and behavioural change supports. Complementing these institutional services are community-based interventions, aftercare programmes, peer-led support structures, and reintegration efforts facilitated through public hospitals, NGOs, and centres under the Drug De-Addiction Programme (DDAP). Many rehabilitation settings also incorporate holistic and lifestyle-oriented approaches—such as yoga, mindfulness, nutritional support, life-skills training, and psychoeducation—to address addiction’s physiological, psychological, and social dimensions in an integrative manner. These models fail to account for how substance use functions in trans men’s lives, where alcohol, tobacco, and unregulated testosterone supplements often acquire socio-symbolic significance. In contexts where endocrinological transition is financially or geographically inaccessible, testosterone substitutes can function as masculinising tools, while alcohol and smoking often operate as markers of masculinity, enabling trans men to enter hypermasculine peer groups or deflect scrutiny. Such practices are not reducible to pathology alone; they are survival strategies in environments structured by cisnormativity.

Verbatim Illustrating Gendered Survival in Public Spaces: “ have public fear. When people stare, I feel like they are looking through me, trying to find out what I am. To avoid that, I started drinking and smoking. It gives me courage to walk in public without feeling watched…. I was travelling on a private bus to Bengaluru and needed to use the toilet. I told the driver to stop near a petrol pump or any public restroom. Instead, he stopped on the roadside and told me to urinate there. He didn’t understand that I am a trans man. I somehow sat and managed to urinate, but when I stood up, he kept looking at me through the mirror, suspiciously. I immediately lit a cigarette … and suddenly their eyes softened. It was like they were telling themselves, ‘Oh, he’s a man.’ Only then did they accept me…” — 32-year-old trans man.

When psychiatry pathologises them without recognising their gendered meanings, it risks misdiagnosis, treatment disengagement, and retraumatisation. High dropout rates and mistrust of clinical services are not surprising outcomes when interventions fail to grasp the social and symbolic dimensions of substance use, particularly for those whose practices are tied deeply to gender affirmation.

What passes as “inclusive” care often remains tokenistic and superficial. Rehabilitation centres may advertise LGBTQIA+ inclusivity, yet in practice, gestures such as rainbow branding, superficial inclusion of LGBTQ+ language in program brochures, or the symbolic hiring of transgender staff without adequate training do little to alter underlying cisnormative assumptions. Such practices risk reproducing epistemic violence, universalising cisgender-centric models while ignoring the identity-specific drivers of distress that shape transmasculine substance use. These token gestures tend to prioritise symbolic visibility over structural transformation, reducing inclusivity to a matter of optics rather than meaningful change. The result is an approach that repackages conventional addiction models with surface-level adaptations, while leaving the foundational structures untouched. Far from expanding care, these centres often replicate the very exclusions they claim to correct, producing new forms of alienation under the guise of progress. While some community-based clinics in urban centres have attempted to integrate transgender staff or inclusive language, their impact remains limited without deeper reforms in therapeutic frameworks.

It is therefore essential to reframe substance use not simply as an individual disorder but as a gendered and relational practice shaped by broader social structures. Masculinity in South Asia has long been associated with alcohol and tobacco consumption (16, 17). For trans men, these practices frequently signify both masculinity and belonging, creating pathways into masculinised peer groups that might otherwise remain inaccessible. What psychiatry diagnoses as addiction may simultaneously function as a means of negotiating gender identity, affirming masculine subjectivity, and securing kinship ties. Recognising these dimensions does not trivialise the risks of addiction; rather, it situates substance use within structural, affective, and symbolic contexts that make treatment meaningful. Such a perspective demands that clinicians broaden their lens, treating substance use not merely as an isolated pathology but as an expression of survival, social identity, and gender performance. If psychiatry continues to treat substance use as a purely individual disorder, it will inevitably miss the causes of both usage and relapse among transmasculine people, leaving therapeutic engagement fractured and incomplete.

Addressing these systemic blind spots requires the development of a rigorous transmasculine-inclusive research agenda that can generate knowledge grounded in lived experience. This must include qualitative and ethnographic work that captures life histories, phenomenological accounts, and the narratives of trans men navigating substance use in contexts of marginalisation. Mixed-methods studies, including longitudinal cohort tracking and geospatial mapping of substance use patterns in relation to healthcare access, could illuminate how rurality, caste, religion, and socioeconomic marginality intersect with gender identity to influence health outcomes. Importantly, such research must not be conducted about transmasculine people without their participation. Community-based participatory research (CBPR) that embeds trans-led organisations throughout design, execution, and dissemination is essential to ensure relational ethics and the co-creation of knowledge. By centring the expertise of trans men themselves, research can move beyond observation to collaboration, bridging the gap between lived realities and clinical practice. Without such epistemic shifts, addiction psychiatry will continue to misalign with those it purports to serve.

Research alone, however, cannot deliver change without reform in medical training and professional practice. Current curricula in psychiatry, psychology, and addiction medicine remain largely cisnormative, with transgender health at best an elective and at worst absent altogether. If addiction psychiatry is to become inclusive, it must decolonise and de-cisgender its educational foundations. This requires embedding modules on trans-specific etiological pathways, mandating demonstrable competencies in transgender health for licensure and continuing education, and developing trauma-informed, culturally relevant relapse-prevention strategies tailored to trans men. Without these reforms, clinicians will remain ill-equipped to recognise how substance use interacts with gendered survival strategies, perpetuating retraumatisation in clinical encounters. Transforming psychiatry’s educational frameworks is not simply an academic exercise; it is a moral imperative. Training is the hinge between research knowledge and practice, and unless it is fundamentally restructured, even the best policies will falter when translated into care.

Policy reform is equally crucial for building an equitable system of addiction psychiatry. Mandating disaggregation of transgender health data by gender identity would not only illuminate hidden burdens but also enable the development of differentiated protocols responsive to the distinct needs of trans men. At the same time, India’s expanding digital health ecosystem offers opportunities to reach populations long excluded from care. Telepsychiatry and AI-driven diagnostic tools, when designed with transmasculine users in mind, could provide culturally adaptive, anonymous, and accessible forms of support. These innovations, particularly if aligned with the WHO’s Global Strategy on Digital Health (2020–2025), could bridge geographic and institutional barriers that have historically constrained access. However, the development of digital platforms must involve trans communities from the outset; otherwise, the biases of offline psychiatry risk being reproduced in digital form. Policy and technology must work in tandem with community leadership to build a system that is not only technically inclusive but socially accountable.

International precedents demonstrate that structural transformation is both possible and necessary. Callen-Lorde’s Transgender Health Program in the United States integrates harm reduction with gender-affirming psychiatry, offering a model for how specialised interventions can be embedded within mainstream services. Casa Nem in Brazil shows the value of community-rooted addiction programs that remain culturally relevant while addressing structural inequalities. Peer-led initiatives in South Africa highlight how community leadership and participatory design can dramatically improve engagement, retention, and therapeutic outcomes. Across these settings, addiction treatment for transmasculine and other transgender people diverges from cisgender male–oriented models in several key respects: it explicitly integrates gender-affirming care (including access to hormones and surgical referrals) with addiction treatment; employs trauma-informed, minority stress–aware psychotherapies; prioritises flexible harm reduction over rigid abstinence; and incorporates peer-led, LGBTQ+-affirming groups that validate gender identity rather than treating it as incidental to substance use. Emerging evidence suggests that such integrated, gender-affirming and peer-supported approaches improve retention, therapeutic alliance, and mental health outcomes for transmasculine clients compared to standard, cisnormative addiction protocols. India can learn from these models, but they cannot simply be imported wholesale. The realities of caste, religion, rurality, and socioeconomic precarity make direct transplantation unworkable. Instead, India must adapt such models in ways that are responsive to local social contexts, ensuring that interventions are not only effective but sustainable. Concretely, this would mean embedding gender-affirming assessment and referral pathways within existing de-addiction services; training clinicians in trauma-informed, LGBTQ+-affirmative motivational interviewing and cognitive-behavioural strategies; creating peer-led transmasculine support groups within hospital and community programmes; and tailoring harm-reduction interventions to the specific ways in which alcohol, tobacco, and hormone-related substances function in trans men’s lives. Community-driven approaches are indispensable, as they ensure accountability, cultural grounding, and long-term impact that extends beyond symbolic reform.

Addiction psychiatry in India stands at a critical juncture. To continue with the homogenisation of transgender identities, the reliance on cisnormative treatment models, and the reliance on token gestures of inclusivity is to reproduce exclusion while claiming progress. What is needed is a decisive, transmasculine-led research and policy movement that integrates disaggregated data, trans-specific research methodologies, educational reforms, and gender-affirming models of care. The goal is not simply to “add” trans men into existing frameworks, but to transform psychiatry itself into a discipline that recognises substance use as both a health issue and a gendered, relational practice. Unless India undertakes this shift, its addiction psychiatry risks replicating exclusion under the guise of inclusivity, leaving health equity unfulfilled. A psychiatry that is truly inclusive must see transmasculine lives not as marginal anomalies but as central to the definition of equitable mental health. Only then can the discipline fulfil its ethical responsibility and reclaim its commitment to justice in practice.
